# Comparative characterisation of different types of Gafchromic films for radiotherapy use

**DOI:** 10.1007/s13246-025-01596-0

**Published:** 2025-07-14

**Authors:** Tarafder Shameem, Nick Bennie, Martin Butson, David Thwaites

**Affiliations:** 1 Northern NSW Local Health District, Lismore, NSW Australia; 2 Institute of Medical Physics, School of Physics, University of Sydney, Sydney, NSW Australia; 3 EPA, Sydney, NSW Australia

**Keywords:** Radiotherapy dosimetry, Radiochromic film, Gafchromic, EBT3, EBT4, EBT-XD, MD-V3, EPSON scanner, Film dosimetry

## Abstract

Different types of Gafchromic films, for radiotherapy use, are recommended for different dose ranges. Ashland Specialty Ingredients has aimed to continuously develop its products to improve their practical application. Thus, EBT3 was replaced by EBT4, intended to provide better signal to noise ratio (SNR); while MD-V3 was introduced for use at higher dose ranges, in addition to EBT-XD. At present there are limited studies on MD-V3. This study aimed to investigate some relevant characteristics of EBT4 and MD-V3, compared with those of EBT3 and EBT-XD. The parameters investigated were dose response, optical density change with post-irradiation time, orientation effect, signal to noise ratio, polarisation, and lateral response artefact (LRA). EBT4 is similar to EBT3 however it provides better SNR and larger response change with post-irradiation time. EBT-XD and MD-V3 are recommended by the suppliers for high dose range, although the sensitivity curves show that EBT3 and EBT4 could also be used for relatively high dose ranges. All films have orientation effects, with EBT3 the worst. An important characteristic of MD-V3 is that the LRA remains similar, irrespective of delivered dose. These comparative characteristics are intended to be informative for clinical practice involving Gafchromic film use in high dose therapy applications. Recommendations from this study are to use EBT4 for dosimetry in lower-dose applications, provided that both calibration and clinical timings post-irradiation are kept similar, while MD-V3 is the preferred film for high-dose procedures.

## Introduction

Gafchromic EBT-series films, by Ashland Specialty Ingredients, G.P., Bridgewater, NJ, USA, are the most popular radiochromic films used in radiotherapy applications. EBT3 film has, been used widely for patient QA for standard dose ranges, until recently when it has been replaced by EBT4. Like any other dosimeter, EBT film dosimetry systems also come with some drawbacks, such as orientation effects and lateral response artefacts (LRA), which result from the film itself and from the scanner used to read the film. Previous studies explained how the rod-like active ingredients of radiochromic films contribute to these effects, which increase with increasing dose of radiation delivered to the film [1, 2]. Dose dependent increase of these effects results from the bonding of the monomers into polymers. Ashland has continuously worked on improving the products to overcome the film-dependent drawbacks. Thus, they introduced EBT-XD in 2015 with shorter active components for use with higher doses and most recently introduced MD-V3, also for higher doses. These two film types, have useful application for dosimetry of high dose patient plans, e.g. for SBRT (Stereotactic Body Radiation Therapy) and SRS (Stereotactic Radiosurgery) radiotherapy. Ashland discontinued EBT3 recently and introduced EBT4, which has the same active ingredient as EBT3 but with different active fluid [3], which resulted in improved signal to noise ratio. Table 1 collates the information given in product brochures [4–7] produced by Ashland.

**Table 1:** Physical properties of different Gafchromic films

Film	Active layer Thickness	Total Thickness	Size	Dynamic Dose Range	Uniformity
EBT3	28 µm	278 µm	8 × 10 inch	0.1 to 20 Gy	± 2%
EBT4	28 µm	278 µm	8 × 10 inch	0.2 to 10 Gy* (optimal dose range)	± 2%
EBT-XD	25 µm	275 µm	8 × 10 inch	0.1 to 60 Gy	± 2%
MD-V3	10 µm	260 µm	5 × 5 inch 8 × 10 inch	1 to 100 Gy	± 2%

^*^In Ashland’s specifications only the optimal dose range is quoted for EBT4 which is up to half of EBT3’s dynamic dose range. This may influence the quoted improved signal to noise ratio which in turn results in improved uncertainty [3, 5]

For dosimetry above 10 Gy Ashland recommends EBT-XD, which has active ingredient lengths (2–4 µm) much smaller than that of EBT3 (15–20 µm) [8] and EBT4. The newest product is MD-V3, which has a significantly extended high dose range compared to the EBT films, however, there is little information available on this film. MD-V3 is supplied in two different standard sizes, 8 inch × 10 inch and 5 inch × 5 inch, and can also be ordered in other customised sizes. In this study, only the 5 inch × 5 inch size films were available. Orientation effects are a well-documented limitation of all radiochromic films. It was anticipated that advancements in film composition would mitigate this issue for MD-V3. The primary cause of the dose dependent variation of the orientation effect is the bonding of active ingredients upon irradiation. Communications with Ashland confirmed that specialised bond-retarding methodology was used to prevent adjacent active ingredients from forming intermolecular bonds upon irradiation (Butson – Private communication – June 2022).

The purpose of this work is to compare various characteristics of these four types of Gafchromic film to provide informative data for practical radiotherapy dosimetry applications. The following parameters were compared:

### Dose response

Though all these four types of film use the same active ingredients, the size of the crystals of EBT-XD is different from that of EBT3 and EBT4, which have the same crystal size [3]. Little specific information is available for MD-V3. The active layer composition is different for all four film types which is apparent based on the colour of the films, which have different dyes in this layer. Because of these changes, the dose responses are different. Due to these differences in the active layer the dynamic range of each type of film is different. This study compares the dose response of 6 MV X-rays and 15 MV X-rays for all four types of film in all three colour channels. Film sensitivity as a function of dose is calculated using film dependent dose-pixel value curves as discussed in the Methods section.

### Change of optical density with post-irradiation time

Following irradiation, radiochromic films continue to darken over time. After a certain period, the rate of colour change decreases significantly, reaching a state that may be considered relatively stable, less than 1% per day. Various studies [1, 5, 9–11] have provided recommendations regarding the optimal waiting period before scanning, to minimize the time-dependent variability in dose response. These have been determined primarily for EBT3 and earlier generations of radiochromic film. This study aims to determine the stabilization time for the four film types, presenting a direct comparative analysis in a single graphical representation.

### Orientation effect

This is a well-known effect in radiochromic films, with quantitative studies and theoretical explanations given in a number of publications [1, 2, 12], mostly for EBT3 and older films. As the active monomers create polymers upon irradiation, this effect increases with dose. There are few studies on EBT-XD [8, 13–15] but no study known to the authors on MD-V3 or on EBT4. Given EBT4 has the same active ingredients as EBT3, it is expected that it may be similar in its behaviour. This study compares the percent change in pixel values for two orientations over six different dose levels.

### Signal to noise ratio

The main supplier-stated improvement of Gafchromic EBT4 film over EBT3 is the improved signal to noise ratio (SNR) [3, 16]. This study investigates this SNR improvement for EBT4, as well the SNR of MD-V3 against EBT-XD, as these two films are recommended for the same high dose range.

### Polarisation

There are a number of studies [2, 17–19] of sources of polarisation in radiochromic film dosimetry systems, which in turn contributes to both orientation and LRA effects. This study compares the change of pixel values due to film-induced light polarization with respect to dose for all four types of films in all three colour channels.

### Lateral response artefacts

A significant challenge in radiochromic film dosimetry is the lateral response artefact (LRA), which refers to variations in pixel values across the film, depending on the position relative to the scanner's centre. In this study, the LRA effect was evaluated for all three colour channels across the four different film types.

## Methods

An Elekta Synergy linear accelerator (Elekta AB (publ), Stockholm, Sweden) was used for all film irradiations, while hand gloves were worn for film handling and processing. Film scanning was performed on an EPSON V800 (Seiko Epson Corp, Nagano, Japan) scanner using the following settings.

- Mode: Professional
- Document Type: Film (with Film Area Guide)
- Film Type: Positive Film
- Image Type: 48-bit colour
- Resolution: 75 dpi
- No Colour Correction was applied

The scanned images were saved as *.tiff (tagged image file format) which were read and separated into three colour (RGB) channels, each of which were analysed using ImageJ V1.49 software [20].

### Dose response

Samples of all four types of film were cut into small pieces of 4 cm × 4 cm and irradiated for doses of 0 Gy, 2 Gy, 5 Gy,10 Gy, 20 Gy, 40 Gy and 100 Gy for 6 MV and 15 MV photon beams. The film pieces were placed in plastic water in a 10 cm × 10 cm field at 10 cm depth, 100 cm SSD with 10 cm backscatter. The film pieces were left in the box for 24 h before scanning. A ROI of 1 cm × 1 cm was drawn at the centre of the image and mean pixel values were calculated. The results were tabulated and plotted as pixel values vs dose. Sensitivity curves were calculated using the first derivative of each dose response curve, defined by the slope of dose (D) and pixel value (PV), using Eq. (1), and plotted against dose delivered.

1 $$\frac{\partial PV}{\partial D}=\frac{PV2-PV1}{D2-D1}$$

### Change of optical density with post irradiation time

Four film pieces of 4 cm × 4 cm, one each of the four types of film, were irradiated with 5 Gy using the same settings as described in the Dose Response section. The films were scanned just after irradiation (0 h) and then every hour for the next 12 h and then after 24 h, 48 h, 72 h and 100 h. The mean pixel values of a 1 cm × 1 cm ROI at the centre of the images were calculated in ImageJ. The values were tabulated and plotted as pixel values normalised to 0 h against time.

### Orientation effect

Films were irradiated for 0 Gy, 2 Gy, 5 Gy, 10 Gy, 20 Gy and 30 Gy. The film pieces were marked to keep track of orientation. As the landscape orientation of Gafchromic films is known to result in higher pixel values [12, 21] and MD-V3 is supplied square in shape, the orientation that gives the higher pixel value for MD-V3 was considered as landscape. Scanned images were read in ImageJ and mean pixel values of a 1 cm × 1 cm ROI were recorded for all film types for all dose levels for all three channels. The films were rotated 90^0^ and the procedure was repeated. The difference of pixel values of the two orientations was calculated for each film in all three colour channels and normalised to the first orientation (portrait) and plotted against dose levels.

### Signal to noise ratio

The same film pieces as used for dose response investigations were also used for this investigation. ROIs of 50 pixels × 50 pixels were drawn at the centre of each film pieces in ImageJ and mean pixel values and standard deviations were recorded. Signal to noise ratios (SNR) were calculated as.

2 $$Signal to noise ratio(SNR)=\frac{PV}{\sigma }$$

where PV is the mean pixel value of the ROI and σ is the standard deviation of those pixel values.

### Polarisation

**Fig. 1:**
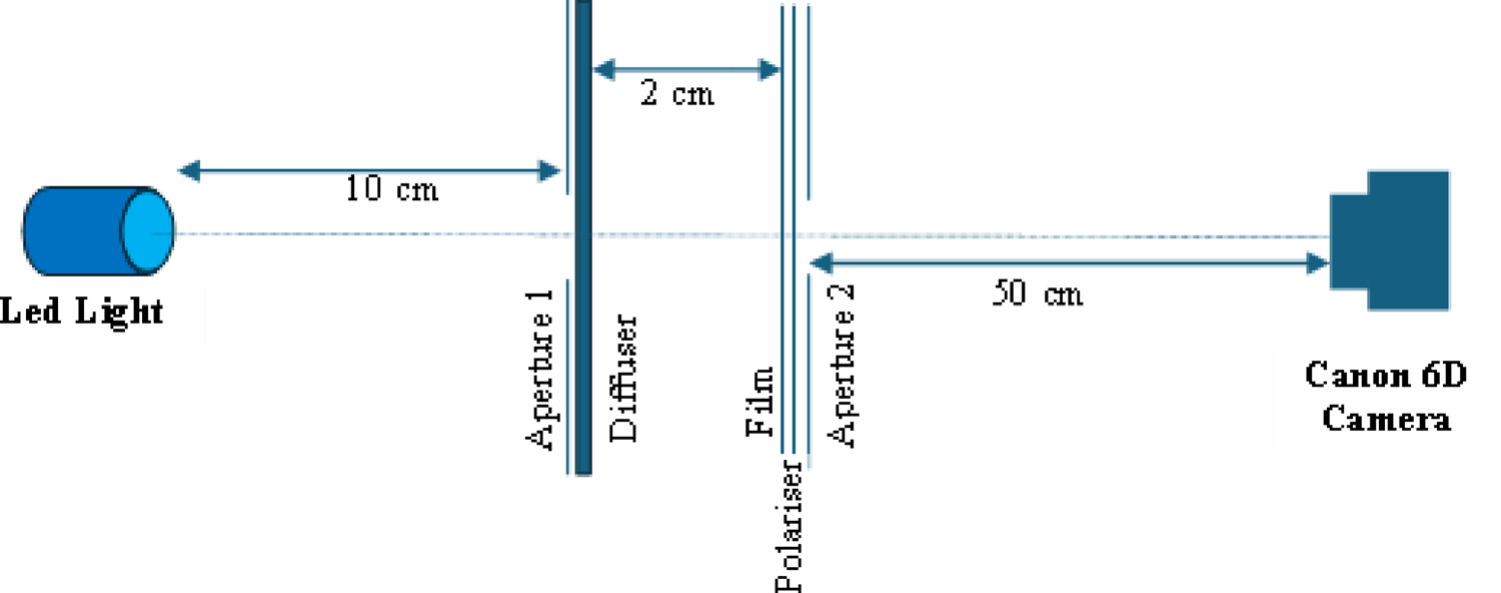
Experimental set up for polarisation measurement

Figure 1 shows the setup for the investigation of film-induced polarisation caused by the four types of films used in this study. A Canon 6D (Canon Inc., Tokyo, Japan) camera was used with Canon 24–105 mm lens, having focal length fixed at 50 mm. The distances between different components are shown in the figure where aperture 1 and diffuser are touching each other, as are film, polariser and aperture 2. The size of aperture 1 and aperture 2 are a 6 cm radius circle and a 4 cm × 4 cm square respectively. Each type of film was irradiated for 0 Gy, 2 Gy, 5 Gy, 10 Gy, 20 Gy and 30 Gy. For each film, two photographs were acquired before and after a 90^0^ rotation of the linear polarizer. Photos were captured in Canon raw format *.CR2 and converted to *.tiff using the Canon software, “Digital Photo Professional”. These were then read and separated into the three colour channels and the mean pixel values found for the image of aperture 2 using ImageJ V1.49 software. The difference between mean pixel values of the two polariser positions for each film was recorded, normalised to the mean pixel values of the first orientation of linear polariser and plotted against the dose values. The LED light source was tested for any inherent polarization by taking two photos with no film, showing that the difference of mean pixel values was negligible, 0.01%, -0.06% and -0.23% in red, green and blue channels respectively.

### Lateral response artefacts

The EBT3, EBT4 and EBT-XD films, which are 20.3 cm × 25.4 cm in size, were cut along the short side to make 3 cm × 20.3 cm strips. For MD-V3 films, only 5 cm × 5 cm pieces were available for this study. These films came with a mark (cut) on one corner of each film piece to mark orientation. They were cut into 3 cm × 5 cm strips and this orientation was marked on all strips. The film strips were placed in plastic water of 30 cm × 30 cm in size and irradiated at 90 cm SSD at 10 cm depth with 10 cm backscatter. A 6 MV photon beam was used to irradiate to 500 MU which corresponds to 5.65 Gy in a 40 cm × 40 cm field size. The film strips were kept in the box for 24 h before scanning. In ImageJ an average profile was generated across each film for each combination using a rectangular ROI of the whole film strip, but cropped 1 mm in from the film edge. The average profiles for each dose level and each colour channel were normalised to the mean of the central 20 data points for analysis. There is a small beam flatness variation over the films at irradiation in these conditions, however the profiles were not corrected for this, since they were only to be compared in a relative manner and this small variation would be common to all. The normalised pixel values were plotted against distance from the centre of the images.

## Results

### Dose response

**Fig. 2:**
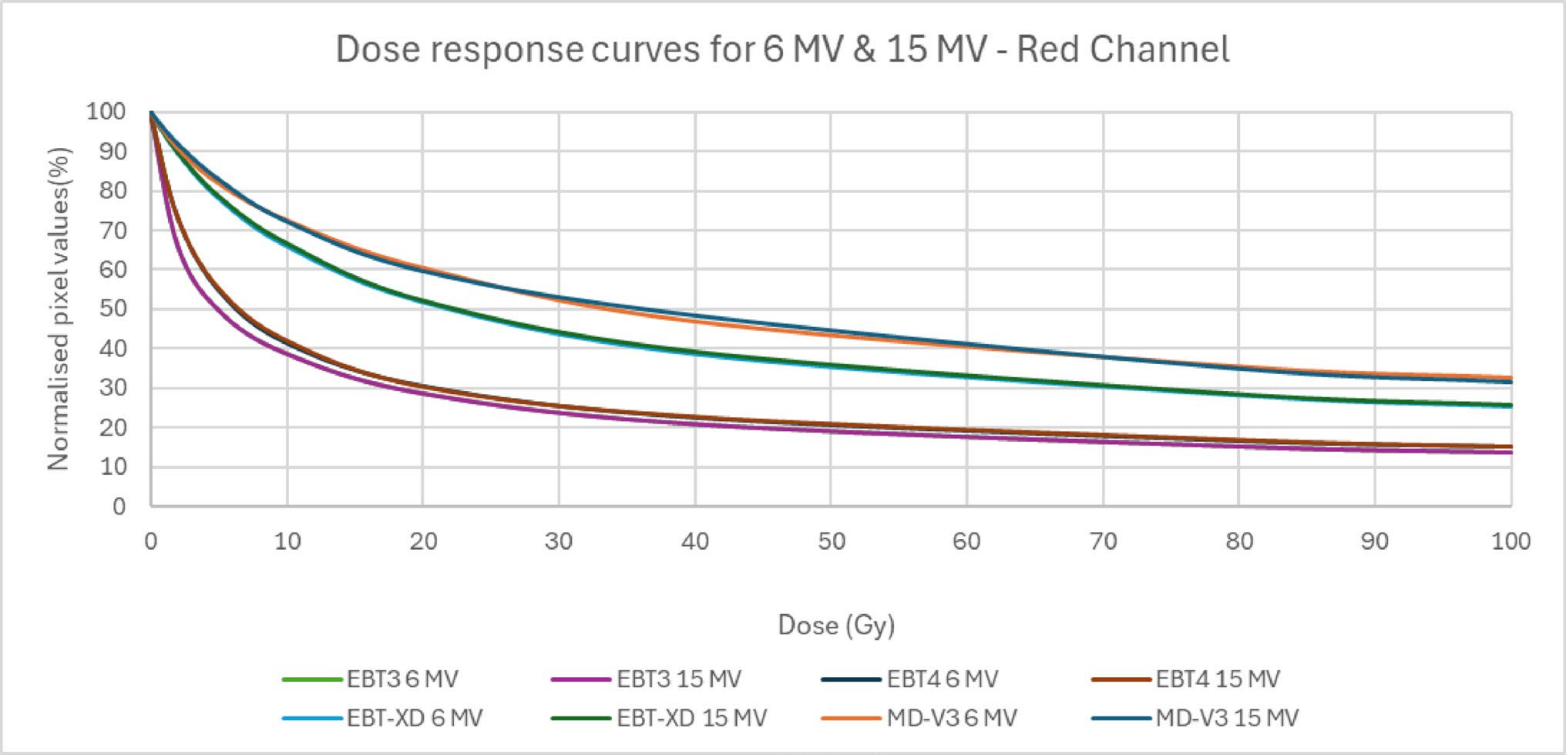
Dose response curves for 6 MV and 15 MV for red channel

Figure 2 presents pixel values normalized to an unirradiated (0 Gy) reference film as a function of delivered dose for 6 MV and 15 MV photon beams in the red channel. The response of EBT3 and EBT4 films is highly similar, while MD-V3 exhibits a similar dose response, though having a little flatter curve, to EBT-XD. No significant differences are observed between the 6 MV and 15 MV dose–response curves for each film type. Although the 6 MV and 15 MV curves for MD-V3 show slightly greater deviation compared to the other three films, the differences remain within the calculated uncertainty, expressed as the standard deviation as a percentage of the mean, which is approximately 0.7% for the selected region of interest (ROI). Other colour channels also showed very similar trends.

**Fig. 3:**
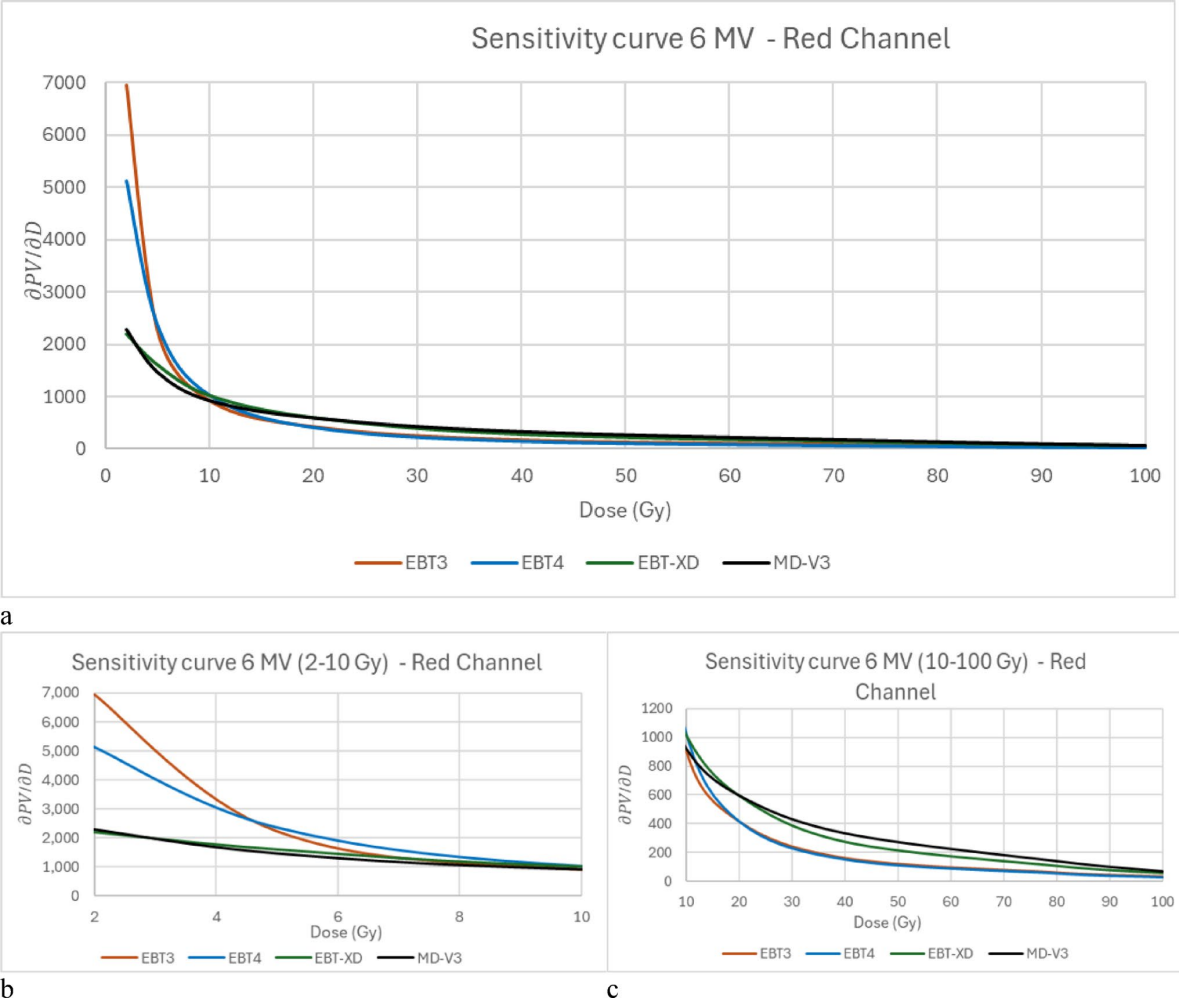
Sensitivity curves of 6 MV for red channel in (**a**) and the same graph divided into two for 0–10 Gy in (**b**) and for 10 – 100 Gy in (**c**)

Figure 3a shows film sensitivity as a function of dose derived from the corresponding dose response data presented in Fig. 2. It shows the change of slope of the dose response curves with respect to dose. The large scale of the y-axis can obscure the differences between the films. Therefore zoomed-in versions, which are presented as 3b and 3c varies the scale to better show the differences. Figure 3b is from 2–10 Gy and Fig. 3c is from 10 to 100 Gy. As expected, in the lower dose region (up to 10 Gy), EBT3 and EBT4 are more sensitive in change of pixel values to change of dose than are EBT-XD and MD-V3, which are quite similar to each other. Above 10 Gy, the sensitivity of EBT-XD and MD-V3 is better than that of EBT3 and EBT4, both of which can still be useful up to 40 Gy.

### Change of optical density with post irradiation time

**Fig. 4:**
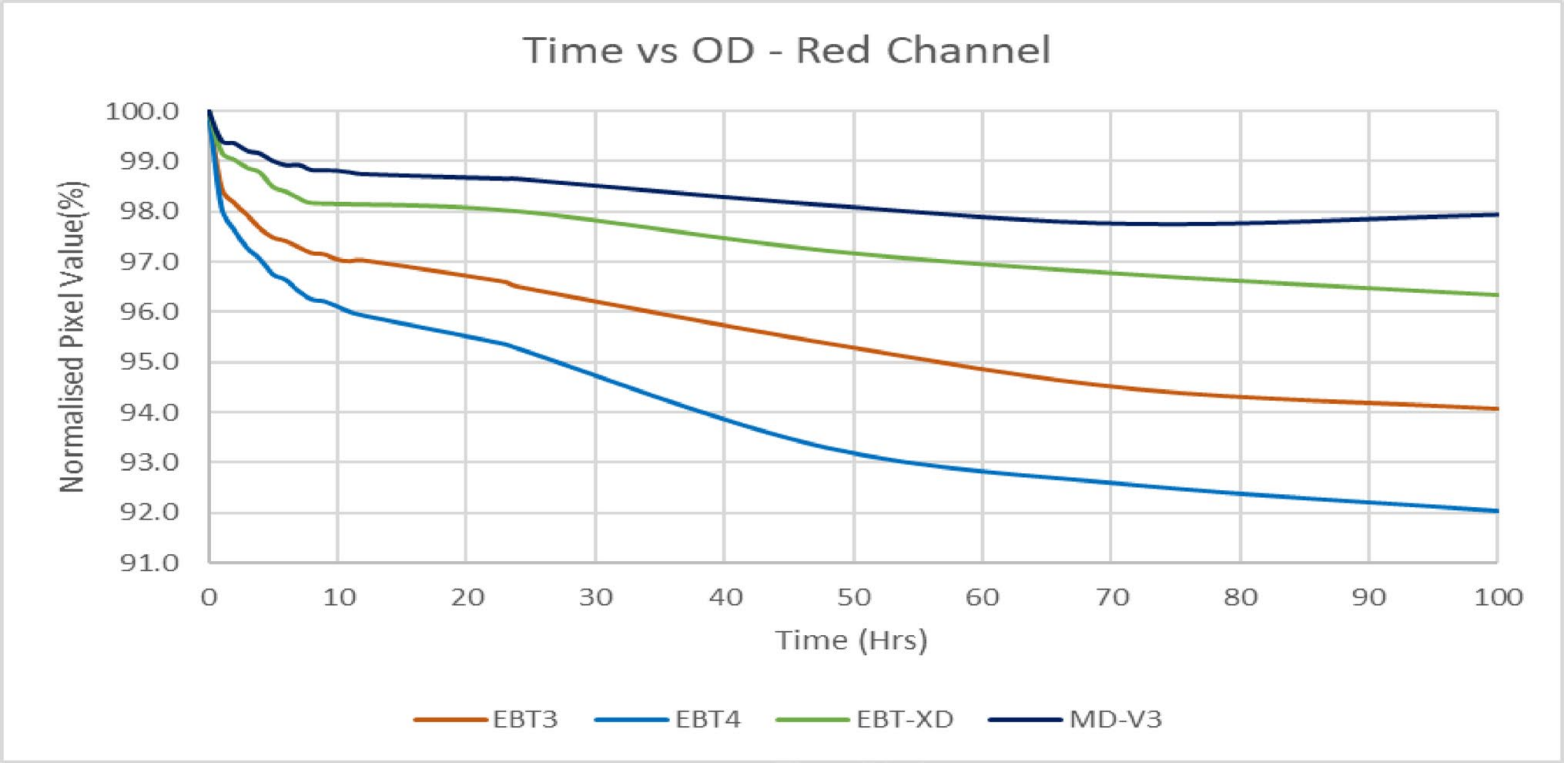
Normalised pixel values with respect to time after irradiation

Figure 4 shows the change of pixel values, normalised to 0 h (just after irradiation) with respect to time in hours in the red channel. EBT4 film has the steepest change of pixel values from the first scan. MD-V3 stabilised after about two hours and all three other films kept darkening to some extent. EBT3 and EBT4 show about 2% and 2.5% drop of pixel values after 2 h, about 0.2% and 0.3% drop per hour in the next 10 h and then 1% and 2% per day respectively. EBT-XD and MD-V3 darkening stabilises much faster, having a drop of pixel values by 1% and 0.6% after one hour, about ≤ 0.1% per hour up to 10 h and about ≤ 0.5% per day after that. Other colour channels also showed very similar trends.

### Orientation effect

**Fig. 5:**
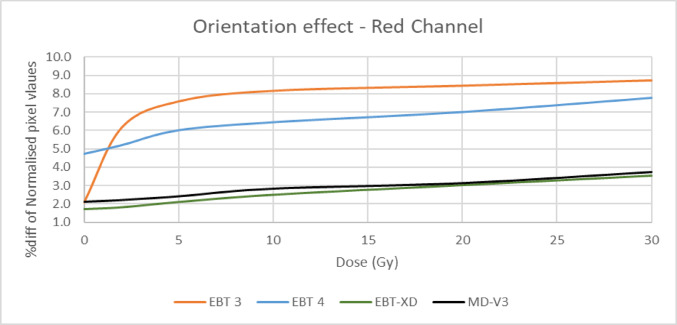
Change of pixel value due to orientation change with respect to dose

Figure 5 shows the change of pixel values due to change of orientation from portrait to landscape plotted against dose for the red channel. All films showed an orientation effect even at zero dose, with EBT4 having the biggest effect of about 5% at zero dose whereas the other three had about 2%. For EBT3 the effect increased steeply up to 5 Gy while the others showed less pronounced changes. MD-V3 and EBT-XD show, but smaller, effects, statistically significant between the two orientations, but in agreement within the statistical errors of the measurements for the two film types. Other colour channels also showed very similar trends.

### Signal to noise ratio

**Fig. 6:**
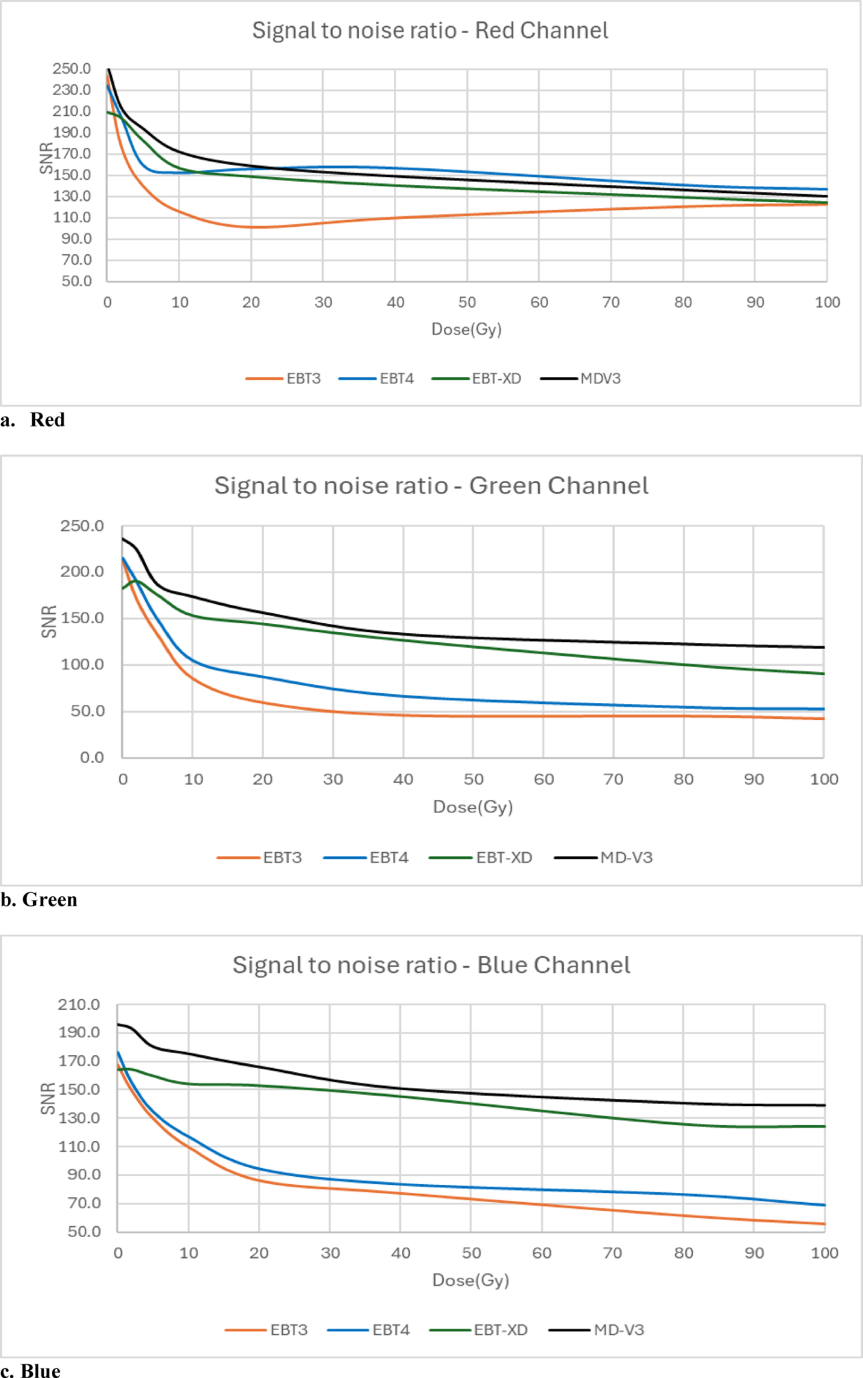
SNR of all four film types in (**a**) red, (**b**) green and (**c**) blue channel

Figure 6a, b and c show SNR with respect to dose from 0 to 100 Gy for red, green and blue channels respectively. SNR of EBT4 is higher than that of EBT3 at all dose levels in the red channel. The other two channels do not show exactly the same behavioural trends with dose, but still SNR is better at all dose levels. MD-V3 shows slightly better SNR than that of EBT-XD at all dose levels in all colour channels.

### Polarization

**Fig. 7:**
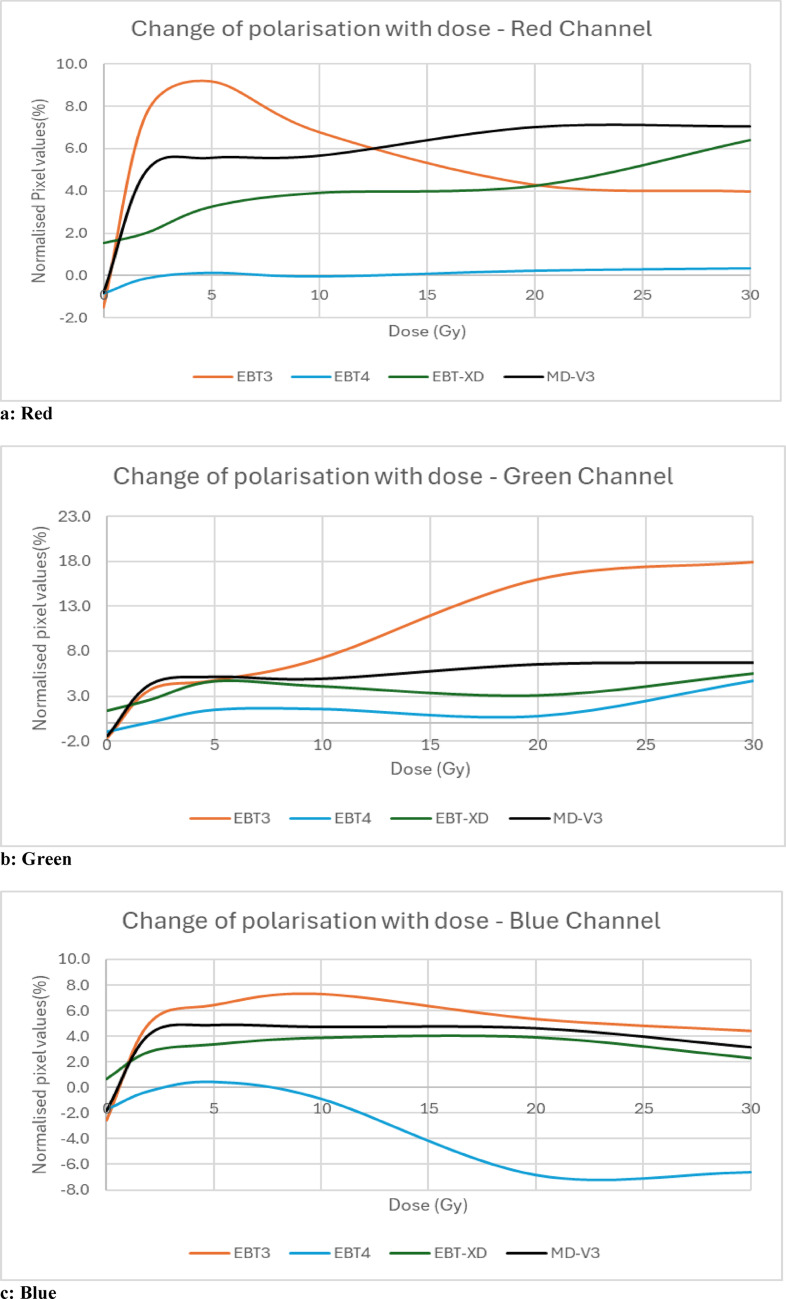
Change of pixel values because of polarisation with respect to dose in red channel (**a**) green, channel (**b**) and blue channel (**c**)

Figure 7a, b, and c illustrate the variation in pixel values between the two polariser positions, normalized to an unirradiated (0 Gy) reference film, as a function of dose for the red, green, and blue colour channels, respectively. While each colour channel exhibits slightly different behaviour, almost all follow a similar general trend: an initial increase up to a certain dose threshold varying across different films and colour channels, followed by either stabilization or a smaller further increase. Exceptions are observed in the red channel for EBT3, where the pixel value decreases after reaching a peak at 5 Gy and in the blue channel for EBT4. Additionally, in the red channel, responses continue to increase at a relatively higher rate in the region of 0—5 Gy compared to the other channels.

### Lateral response artefact

**Fig. 8:**
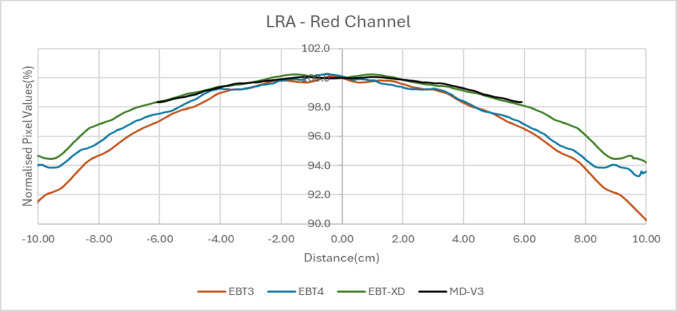
Change of pixel values from central axis for all four film types in red channel for 5 Gy

Figure 8 presents the variation in pixel values in the red channel, normalized to the film centre, across the short side of the films in portrait orientation. For the MD-V3 films the strips were cut consistent with this, using the assigned orientations discussed in the orientation effects investigation. The dose profile curvature of EBT-XD and MD-V3 is similar, while EBT3 exhibits the most pronounced lateral response artefact (LRA), with EBT4 demonstrating an intermediate effect. Other colour channels also showed very similar trends.

## Discussion

### Dose response

Consistent with findings from product brochures and previous studies [3, 16, 22–25], the difference in energy dependence between 6 and 15 MV photon beams is negligible for all four film types. While all three colour channels were analysed, only the red channel results are presented here, as the green and blue channels exhibit similar trends, showing no significant difference between 6 and 15 MV beams. The dose–response curves for EBT3 and EBT4 are very similar at the dose levels investigated in this study, differing by no more than 2%, which aligns with previous investigations [3]. The manufacturer recommends dose ranges of up to 60 Gy for EBT-XD and up to 100 Gy for MD-V3. However, an analysis of the first derivative (sensitivity curve) of the 6 MV dose–response curves reveals that there is not much difference between EBT3 and EBT4, or between EBT-XD and MD-V3 in terms of change of slope with dose beyond 10 Gy, indicating no significant calibration advantage in using MD-V3 over EBT-XD. In addition, this implies that although EBT3 and EBT4 are less sensitive in the higher dose ranges than the other two film types, they could still be used up to around 40 Gy. The manufacturer's brochures provide dose–response curve data only up to 22 Gy for EBT3, EBT4, and EBT-XD, and up to 100 Gy for MD -V3. Abe et al. [26] previously reported that EBT3 exhibits saturation beyond 100 Gy.

### Change of OD with Time

The results of the red channel are presented here; as the green and blue channels follow similar trends. EBT-XD and MD-V3 exhibit substantially faster stabilization, with pixel value changes of approximately 1% between 2 and 24 h post-irradiation. In contrast, EBT3 undergoes a ~ 2% change over the same period and continues to change by approximately 1% every subsequent 24 h. EBT4 demonstrates more prolonged darkening, stabilizing only after 48 h. To minimize the impact of post-irradiation darkening the time interval between irradiation and readout for both calibration curve generation and patient QA image acquisition must be kept similar when using EBT4 in clinical applications. This is a significant consideration for clinical use of the newer EBT4 film.

### Orientation

The results of the red channel are presented here; the green and blue channels follow similar trends. Previous studies [12, 21] showed EBT film has an orientation effect, which stays the same with increasing dose. However, both investigations were carried out only up to 3 Gy. The orientation effect was investigated for EBT-XD and EBT3 films by Khachonkham et al. [22] but not given any numerical values with respect to dose. They presented two calibration curves of portrait mode and landscape mode. The curves showed increasing deviation until about 5 Gy for EBT3 and until 15 Gy for EBT-XD, after which the difference remained the same. The current study also found a similar trend for EBT3 but for EBT-XD the difference kept increasing, by about 1% from 15 to 30 Gy. The uncertainty, on the values, calculated as the standard deviation as a percentage of mean pixel value of the selected ROI, is 0.7%. The measured orientation effects are statistically significant for all films.

### Signal to noise ratio

Guan et al. [16] reported SNR for red channel and Palmer et al. [3] reported SNR for red and green channels for EBT3 and EBT4 for up to 10 Gy. The results for red channel of this current investigation are in line with these two previous red channel results, showing that SNR for EBT4 is better than for EBT3 at all dose levels. The green channel results are also similar to those reported by Palmer et al. [3], but the raw pixel values are not exactly the same. Shameem et al. [27] reported that pixel values produced by different scanners are different for the same film. Palmer et al. [3] used an EPSON 12000XL scanner, whilst an EPSON V800 scanner was used in this study. The difference in raw pixel values for similar dose levels may result from the use of different scanners in these two investigations.

MD-V3 exhibits a higher signal-to-noise ratio (SNR) than EBT-XD across all dose levels and in all three colour channels. This slight improvement may be attributed to the different dye formulation used in MD-V3, although this was neither reported nor mentioned in the product brochures [7]. In contrast, the EBT4 product brochure [5] explicitly highlights its SNR enhancement as a key advantage, as compared to EBT3.

### Polarization

Previous studies have investigated light polarization effects in various Gafchromic films, including EBT, MD-55 [17], EBT-HS [28], EBT [18], EBT3 and EBT-XD [8]. With the exception of EBT-HS, all Gafchromic films have been shown to induce light polarization, with the effect increasing as a function of dose.

The current study employed a methodology similar to that of Schoenfeld et al. [8], utilizing a camera instead of a scanner to analyse light polarization in Gafchromic films. This approach was adopted based on our recent findings [29] indicating that the mirror systems in scanners can themselves introduce polarization artefacts, which contribute to LRA and orientation effects and which are likely to vary with scanner [29, 30]. We previously suggested [29] that imaging systems with modified designs using only one mirror (or ideally none) would reduce the scanner-induced polarisation effect.

Results demonstrated that light polarization patterns vary across different colour channels, particularly in EBT3, where each channel exhibits a distinct response. Further investigation is currently underway to better understand these variations. Additionally, EBT4 shows a notably different response in the blue channel. In contrast, EBT-XD and MD-V3 exhibit more consistent behaviour across all three colour channels, characterized by an initial increase in polarization followed by stabilization.

These findings generally align with previous studies, confirming that, except for EBT-HS, light polarization induced by Gafchromic films increases with dose when compared to an unirradiated film.

### Lateral response artefact

Initially, for the investigation of lateral response artefacts (LRA) in this study, the films used were exposed to 5 Gy. Previous studies [8, 14, 15, 19] have examined the LRA effect on EBT3 and EBT-XD, revealing that the lateral profile for EBT-XD is flatter than that of EBT3 at the same dose, with the improvement attributed to the smaller size of the active ingredients [13]. Lewis et al. [14] demonstrated that when the transmitted light intensity through the films is the same, the curvature of the profile remains consistent. Since EBT-XD darkens less than EBT3 for the same dose, due to its smaller size active ingredients, the resulting curvature is flatter.

Given that MD-V3 darkens even less than EBT-XD, it was expected to exhibit a flatter curve. However, the results showed that the profile of MD-V3 was very similar to that of EBT-XD, contradicting previous findings. Therefore, further investigation was conducted to explore this discrepancy, considering the LRA at different doses to the films.

**Fig. 9:**
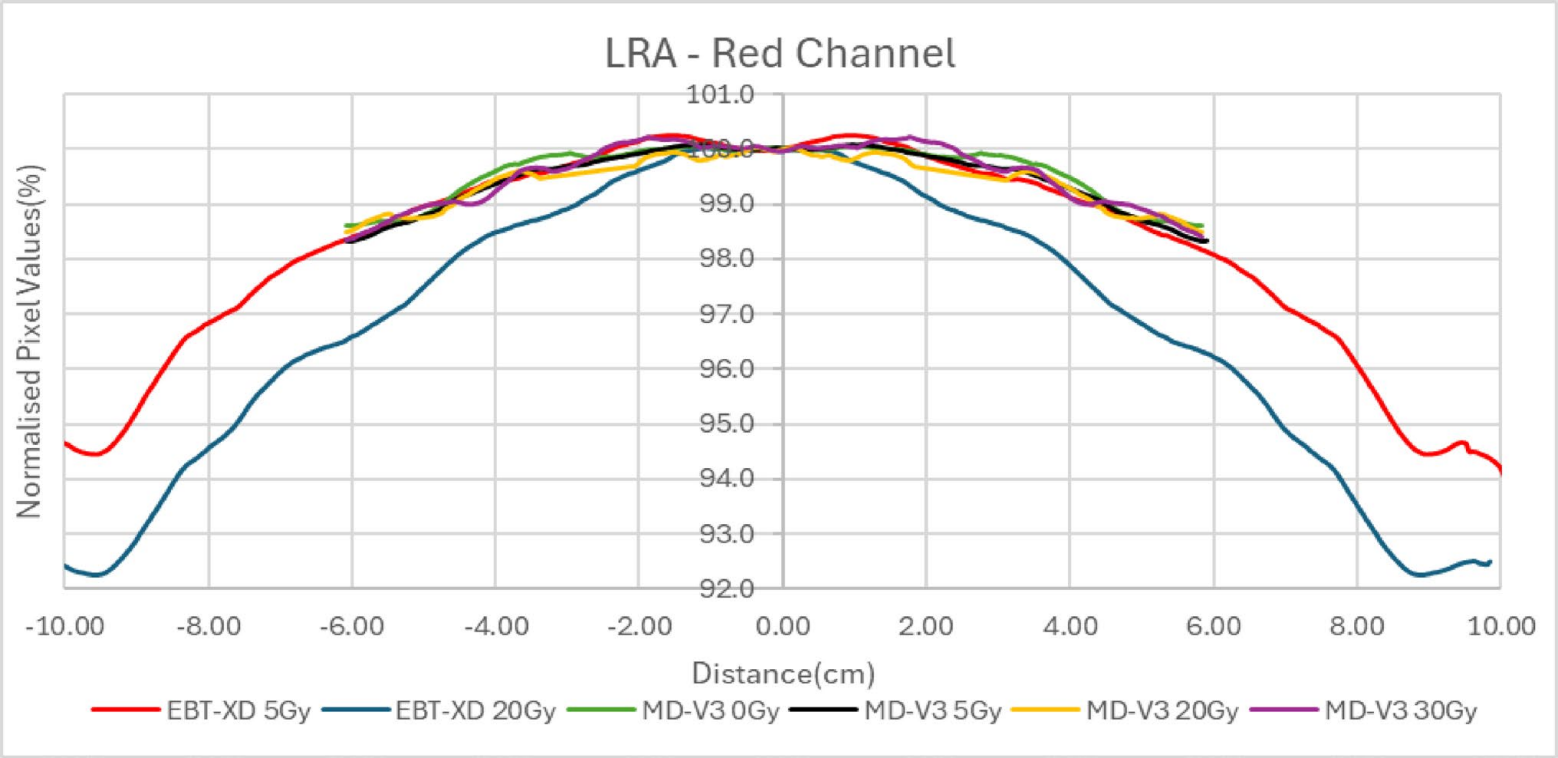
LRA for different dose values of EBT-XD and MD-V3

Figure 9 presents the relative dose profiles for films irradiated with 5 Gy and 20 Gy for EBT-XD, and 0 Gy, 5 Gy, 20 Gy, and 30 Gy for MD-V3. As expected, the LRA effect for EBT-XD increased with dose, with the 20 Gy profile exhibiting more pronounced LRA than that for 5 Gy. However, all profiles for MD-V3 were very similar, showing no significant dependency on dose. This lack of LRA dose dependence in MD-V3, suggests that the dose profile remained constant due to a factor beyond darkening alone. This phenomenon is tentatively attributed to the addition of the bond-retarding ingredients in the active layer formulation, which may inhibit bonding between the monomers of the active ingredients, as discussed in the introduction. This lack of increasing LRA with dose gives MD-V3 an advantage over EBT-XD for clinical applications.

## Conclusions

This study provides a comprehensive characterization and comparison of four types of Gafchromic films, EBT3, EBT4, EBT-XD and MD-V3, investigated mainly on an Epson V800 scanner, selected as one widely used in clinical dosimetry. For measurements on the different films for each part of the study, the scanner and other experimental parameters were kept as consistent as possible between film types to draw out differences due to the films themselves. It may be noted that exact results may differ on other scanners, but that the relative findings between the film types are expected to be similar.

Among these four film types, MD-V3, the newest addition to the Gafchromic family, has not been extensively studied previously, making it a key focus of this investigation. EBT3, which has recently been replaced by EBT4, and EBT-XD are widely used in radiotherapy applications for standard and high-dose ranges, respectively. While EBT3 and EBT4 are recommended for standard dose ranges (up to 10 Gy), and EBT-XD (up to 60 Gy) and MD-V3(up to 100 Gy) for high-dose applications, this study found that EBT-XD and MD-V3 exhibit similar sensitivity curves, indicating that each could be utilized across a broad range of doses. EBT3 and EBT4 have a significant advantage over the other two in low dose ranges (< 10 Gy) in terms of dose response sensitivity but could still be used up to 40 Gy.

Despite initial assumptions that the supplied square shape of the available MD-V3 films might indicate reduced orientation effects, it was observed that its orientation effect is similar to EBT-XD, though much smaller than those seen in EBT3 and EBT4. As expected, EBT4 demonstrated a significantly improved signal-to-noise ratio (SNR) over EBT3. The SNR was also improved for MD-V3 in comparison to EBT-XD, however, this was to a lesser extent than that between EBT3 and EBT4. The major limitation of EBT4 film is the increased post-irradiation darkening which extends beyond 100 h. This can have a significant impact when using EBT4 film clinically. To mitigate this, it is important to maintain a consistent time gap between irradiation and scanning, for both calibration and QA measurement films. Provided this recommendation is adhered to, EBT4 provides more reliable results than EBT3. For MD-V3 film, the most noticeable advantage is the consistent lateral response artifact with increasing dose which was not observed for EBT-XD.

Based on our investigation of EBT3, EBT4, EBT-XD and MD-V3 film, it has been determined that EBT4 is a suitable replacement for EBT3 film, provided consistent post-irradiation analysis times are followed. It is recommended to use EBT4 film for all low dose measurements, while MD-V3 should be utilized for high-dose measurements exceeding 10 Gy. These recommendations are made from consideration of the various effects studied here, ensuring optimal performance of the films with the least need for scanner or software corrections.

## Data Availability

All the data are available from the corresponding author by request.
